# Editorial: Host-microbe immunometabolic chat: a new era of organismal communication

**DOI:** 10.3389/fimmu.2026.1882702

**Published:** 2026-05-29

**Authors:** Marco Gargaro, Francesca Fallarino, Teresa Zelante

**Affiliations:** 1 Department of Pharmaceutical Sciences, University of Perugia, Perugia, Italy; 2 Department of Medicine and Surgery, University of Perugia, Perugia, Italy

**Keywords:** indoles, metabolite, microbiome, multiple sclerosis, SCFA (short chain fatty acid), TME (tumor microenvironment), tryptophan

Microbiota represents a key regulator of host physiology, exerting its multiple functions through a complex network of metabolic, immune, and neuroimmune interactions. Rather than acting locally within the gastrointestinal tract, microbial signals influence distant tissues systemically, shaping immune homeostasis and disease susceptibility at the organismic level. These studies collectively reflect the growing recognition that immune regulation is not confined to a single organ but arises from dynamic interactions among microbial metabolites, host metabolic pathways, and neuroimmune signalling.

The articles collected in this Research Topic provide a comprehensive and integrative view of this emerging paradigm, highlighting how microbiota- and host-derived molecules converge to shape immune homeostasis and disease in several biological settings. A central theme emerging across several contributions is that microbiota composition does not strongly impact physiology and the immune system but mainly serves as a ‘chat’, in which the host and the microbiota communicate through metabolic pathways.

Therefore, the composition of the host microbiota appears to play a role in systemic signalling only if merging with a metabolic molecule. This is true for the paper by Ricci et al., in which a metabolomic profile was executed, identifying microbiota-derived metabolites as systemic signalling molecules in both healthy individuals and patients with relapsing-remitting Multiple Sclerosis (MS), before and after treatment with Ocrelizumab. By looking at the tryptophan metabolites derived from the host and microbes, they found that mainly bacterial indoles are differentially measured in the plasma of MS patients. In particular, Indole-3-aldehyde is significantly higher in untreated MS patients compared with healthy controls or after treatment with Ocrelizumab, suggesting changes in the microbiota during the disease. In addition, melatonin is also increased in untreated MS patients. Melatonin is well described as part of the microbiome metabolism in the gut and is particularly elevated in chronic inflammatory diseases. Therefore, in the paper by Ricci et al., microbes ‘chat’ via tryptophan metabolism ultimately compensating for the host inflammatory status in MS.

Short-chain fatty acids (SCFAs) represent one of the most extensively studied classes of microbial metabolites. In their research article, He et al. search for a wide distribution of host receptors for microbial SCFAs. For instance, they tried to understand how and where the human host responds to SCFAs to establish ‘multiple chats’.

Using 18 public single-cell RNA-seq and single-nuclear RNA-seq data of human and mouse tissues, the authors illustrate the entire atlas of FFAR2/3 distribution. They found that receptors for SCFAs are particularly expressed on fibroblasts and myeloid cells, underlining the importance of the ‘chat’ in inflammation and recovery from tissue necrosis.

Accordingly, microbiota-derived signals also shape immune responses in distal mucosal sites.

Ye et al., in their review, describe how pneumonia may impact intestinal dysbiosis and inversely how gut dysbiosis affects host lung inflammation. The ‘chat’ here is established between the lung and gut *via* SCFAs.

Similarly, Qi et al. describe the complex communication axis between the gut microbiota metabolites – such as polyphenols, vitamins, and indoles – that contribute to the initiation and progression of gout and the release of uric acids in the peripheral blood. In the context of

dysbiosis, an increase in the secretion of lipopolysaccharides, which trigger metabolic endotoxemia and systemic inflammation, exacerbates gout flares. In addition, compromised barrier integrity facilitates the translocation of bacteria and their metabolites into the bloodstream, amplifying inflammation and promoting the deposition of urate crystals, thereby increasing the risk of gout. In addition, the original research article by Wei et al. shows that washed microbiota transplantation restores circulating group 3 innate lymphoid cells, enhances IL-22 signalling, and improves lipid metabolism in hyperlipidemic patients, identifying a microbiota–immune–metabolic axis with direct clinical implications.

A similar metabolic chat is established systemically, affecting the tumour microenvironment, as reported by Hu et al.‘s mini review. This ‘intratumoral chat’ appears particularly interesting now, considering the importance of the intratumoral microbiome and the increasing ability to delineate it technically inside distinct tumours. Therefore, investigating the therapeutic potential of gut microbiota-derived metabolites represents an emerging frontier in precision oncology, presenting new opportunities to improve clinical outcomes of cancer patients. Duan et al. also discuss the importance of metabolites in modulating the tumour microenvironment by focusing on AhR and the ligands that activate this nuclear receptor.

An interesting clinical case of sepsis was reported by Xu et al., where the use of both metagenomics and blood culture was employed, demonstrating the need for novel sepsis biomarkers, eventually based on metabolomics in combination with metagenomics and blood cultures.

In addition to metabolic regulation, emerging evidence highlights a critical contribution of neuroimmune interactions. Liu et al. describe how pathogens can capture nociceptor sensory neurons through TRPV1/TRPA1 activation and CGRP release, leading to suppression of immune function. This mechanism of neuroimmune modulation reflects a larger host–pathogen interaction ‘chat’ and communication strategies from the periphery to the central nervous system. Zhao et al. focus on the role of the microbiome in regulating microglial activation, astrocyte function, and blood–brain barrier integrity, thereby maintaining immune homeostasis within the nervous system. Ataei et al. extend this framework to neuropsychiatric disorders, linking microbiota-derived metabolites and immune signalling to neurotransmitter balance, vagal signalling, and behavioural outcomes. Therefore, it appears that the emergence of novel therapies targeting interconnected immune and neural pathways may also affect the microbiome niches. Hu et al. demonstrate that the Yinhua Gouteng Decoction, a traditional Chinese medicinal formula, has neuroprotective and anti-inflammatory effects in a 3,3′-iminodipropionitrile-induced TD rat model of human TIC disorder. The main effects were on microglial activation and reduced the levels of inflammatory cytokines in the striatum, serum, and colon. Furthermore, the decoction restored gut dysbiosis – particularly by modulating pro-inflammatory taxa – and downregulated the TLR4/MyD88/NF-kB signalling pathway in both the striatum and colon.

Taken together, the multiple ‘chat’ described in this Research Topic converge on a unified conceptual framework in which microbiota, microbiota-derived metabolites, immune responses, and neuroimmune interactions form an integrated regulatory network. Rather than functioning independently, these systems operate as interconnected components of organismal communication, by creating a ‘gut-metabolite-immune axis’, influencing disease susceptibility, progression, disease diagnosis and therapeutic responses.

